# Bundling up the Role of the Actin Cytoskeleton in Primary Root Growth

**DOI:** 10.3389/fpls.2021.777119

**Published:** 2021-12-16

**Authors:** Judith García-González, Kasper van Gelderen

**Affiliations:** ^1^Department of Experimental Plant Biology, Faculty of Science, Charles University, Prague, Czechia; ^2^Laboratory of Hormonal Regulations in Plants, Institute of Experimental Botany, Czech Academy of Sciences, Prague, Czechia; ^3^Plant Ecophysiology, Department of Biology, Faculty of Science, Utrecht University, Utrecht, Netherlands

**Keywords:** actin, root growth, cell elongation, auxin, gravitropism, actin-binding protein, light

## Abstract

Primary root growth is required by the plant to anchor in the soil and reach out for nutrients and water, while dealing with obstacles. Efficient root elongation and bending depends upon the coordinated action of environmental sensing, signal transduction, and growth responses. The actin cytoskeleton is a highly plastic network that constitutes a point of integration for environmental stimuli and hormonal pathways. In this review, we present a detailed compilation highlighting the importance of the actin cytoskeleton during primary root growth and we describe how actin-binding proteins, plant hormones, and actin-disrupting drugs affect root growth and root actin. We also discuss the feedback loop between actin and root responses to light and gravity. Actin affects cell division and elongation through the control of its own organization. We remark upon the importance of longitudinally oriented actin bundles as a hallmark of cell elongation as well as the role of the actin cytoskeleton in protein trafficking and vacuolar reshaping during this process. The actin network is shaped by a plethora of actin-binding proteins; however, there is still a large gap in connecting the molecular function of these proteins with their developmental effects. Here, we summarize their function and known effects on primary root growth with a focus on their high level of specialization. Light and gravity are key factors that help us understand root growth directionality. The response of the root to gravity relies on hormonal, particularly auxin, homeostasis, and the actin cytoskeleton. Actin is necessary for the perception of the gravity stimulus via the repositioning of sedimenting statoliths, but it is also involved in mediating the growth response via the trafficking of auxin transporters and cell elongation. Furthermore, auxin and auxin analogs can affect the composition of the actin network, indicating a potential feedback loop. Light, in its turn, affects actin organization and hence, root growth, although its precise role remains largely unknown. Recently, fundamental studies with the latest techniques have given us more in-depth knowledge of the role and organization of actin in the coordination of root growth; however, there remains a lot to discover, especially in how actin organization helps cell shaping, and therefore root growth.

## Introduction: The Actin Cytoskeleton and Primary Root Growth

The root system is an essential part of the plant that navigates the soil for water and nutrients. The primary root is the first organ to emerge from the seed, and therefore, its developmental plasticity is of utmost importance. The cytoskeleton is an interconnected filamentous network that is the key in controlling cell shape, rigidity, supports intracellular processes, and consists of microtubules and actin filaments (AF). AF consist of two helical strands of actin monomers that grow from the barbed plus end (compared to the pointed minus end) and rely on a plethora of actin-binding proteins to modify their organization and dynamics. Actin filaments are crucial for cell and tissue growth and participate in a large variety of processes including cell architecture and polarity establishment, signal transduction, cell-cell communication, cell division, and response to pathogens. Furthermore, the actin cytoskeleton is known to be an important player in vesicle trafficking, secretion, and endocytosis as well as endomembrane remodeling. By controlling the growth and division of cell, the actin cytoskeleton provides the means to dynamically respond to intracellular and extracellular stimuli (Mao et al., 2016; Zhu et al., 2016; Maeda et al., 2020) (Reviewed in Paez-Garcia et al., 2018; Li and Day, 2019; Takatsuka and Ito, 2020; Yang et al., 2020). The actin cytoskeleton responds to an extensive amount of stimuli, such as gravity, osmotic stress, pathogens, nutrients, and light (Henty-Ridilla et al., 2013; Leontovyčová et al., 2019) (Reviewed in Li and Day, 2019; Nakamura et al., 2019; Wang and Mao, 2019; Leontovyčová et al., 2020; Zhao et al., 2021). Plant hormones, and especially auxin, have strong effects on the composition of actin filaments (Arieti and Staiger, 2020; Reviewed in Zhu and Geisler, 2015). The actin cytoskeleton has been studied extensively; however, the role that it plays in regulating the growth of the primary root is complex and often yields conflicting data. We hereby review how actin helps root growth. We first discuss how actin filaments organize during root growth. Then, we dissect how individual components of the actin filaments and the actin-binding proteins affect root growth. Next, we describe the evidence on how the hormones controlling growth affect the actin cytoskeleton and finally we discuss how actin affects the root tropisms to gravity and light.

## Actin Organization and Dynamics Correlate with Root Cell Elongation

The main region that sustains primary root growth is the root apical meristem (RAM) through a tight balance between cell division and elongation (Ioio et al., 2008; Reviewed in Takatsuka and Umeda, 2014; Ötvös et al., 2021). The actin cytoskeleton has been connected to root growth through its participation mainly in cell expansion and, secondarily, cell division (Ingouff et al., 2005; Kandasamy et al., 2009; Takatsuka et al., 2018; Arieti and Staiger, 2020). Root meristem cells undergo two steps of rapid cell elongation. The first one occurs in the progression between the apical meristem and the transition zone (TZ), which is triggered by an endoreduplication event. A second fast elongation takes place in the boundary of the transition and elongation zones (EZ), characterized by an increase in vacuolar enlargement accompanied by cell wall loosening and new material deposition (Reviewed in Verbelen et al., 2006; Takatsuka and Umeda, 2014; Barrada et al., 2015). Actin cytoskeleton dynamics have often been connected to root cell elongation. Initial studies in the monocot *Zea mays* pointed out specific actin arrangements depending on the cellular developmental stage in the longitudinal axis of the RAM. Immunolocalization revealed longitudinally oriented, often bundled, Filamentous actin (F-actin) in elongating cells while this disposition was lost during maturation (Baluška et al., 1997; Blancaflor and Hasenstein, 1997).

**Figure 1:**
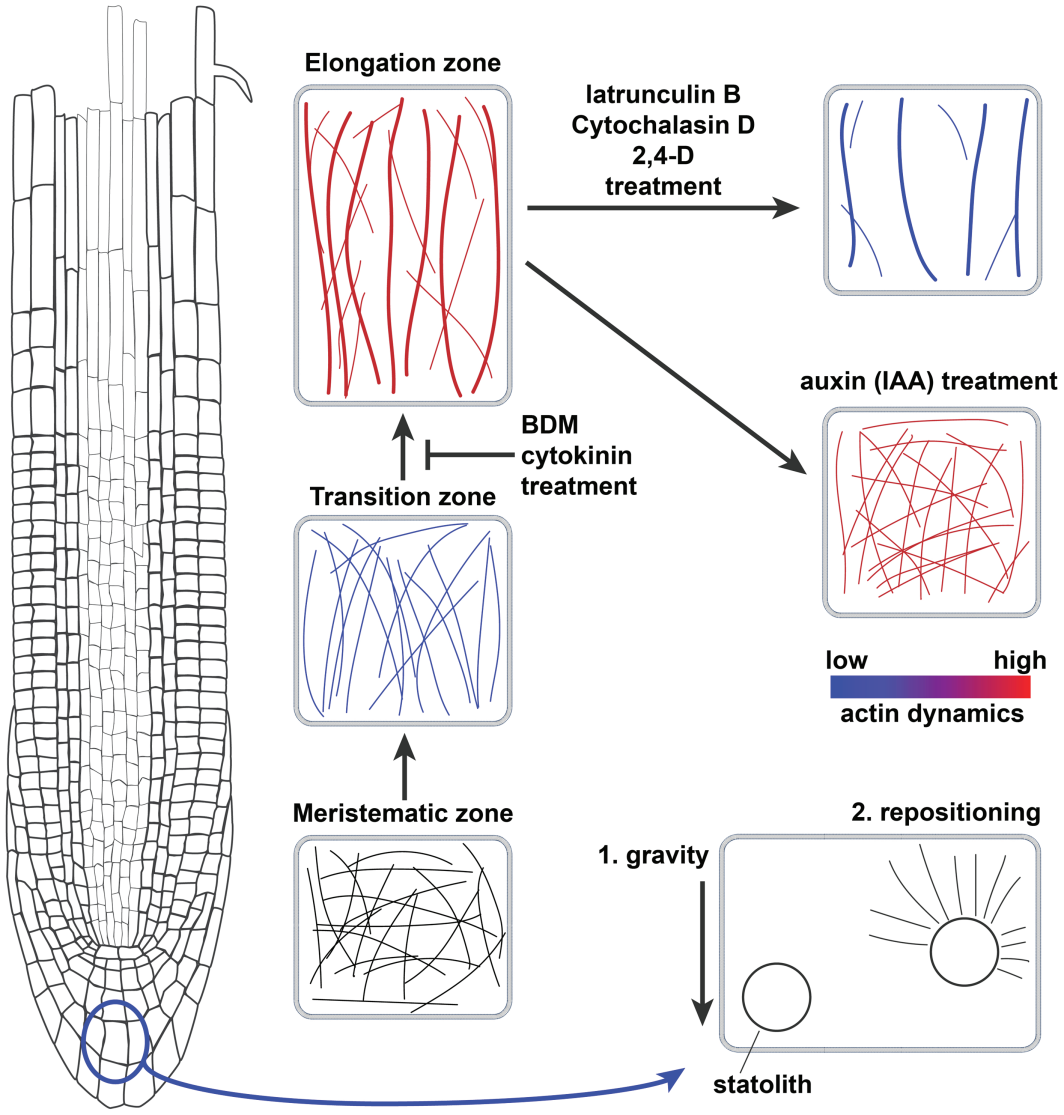
Actin filament network organization in the *Arabidopsis thaliana* root meristem and the effects of drugs, hormones, and gravity. Schematic representation of the actin filament networks in different cell types of the root meristem and the effect of drugs and hormones on the actin network. Thicker lines equal more bundling of actin filaments, while blue and red coloring denotes actin dynamics. Bottom right cell shows the role of actin filaments in repositioning the gravity-sensing statoliths.

Later, more detailed work has been done describing actin cytoskeleton organization and dynamics in epidermal root cells of the model dicot *Arabidopsis thaliana*. There are clear differences between RAM zones (Figure 1); to start with, meristematic cells show a pattern of dense, highly disorganized actin, characterized by relatively low levels of bundling and reduced longitudinal orientation and parallel arrangement of microfilaments (Wang et al., 2004; Arieti and Staiger, 2020). The TZ shows comparable actin filament density to that of the meristem; microfilament orientation remains random but an increase in parallel organization is observed. Actin dynamics in this developmental stage are reduced, although there is an increase in annealing events (Takatsuka et al., 2018; Zou et al., 2019; Arieti and Staiger, 2020). An increase in longitudinal actin filament bundling occurs preceding the second fast growth event during the transition to the EZ (Takatsuka et al., 2018). Cells in the elongation zone show a pattern of a diffuse and less dense actin network with longitudinal bundled arrays that display higher dynamics and longer, faster growing actin filaments (Wang et al., 2004; Dyachok et al., 2011; Jacques et al., 2013; Vaškebová et al., 2018; Zou et al., 2019; Arieti and Staiger, 2020).

As a generalization, there is a correlation between root cell length and the presence of less dense, more bundled and longitudinally oriented actin filaments (see overview in Figure 1). We do not know whether these differences in actin organization are coupled to different roles of actin, or that it simply reflects the differences in cell morphology at different developmental stages. A useful way of correlating changes in actin with the effects on root growth is the analysis of the effects of actin-disrupting drugs on root growth (for a summary of drugs, see Table 1). Actin-stabilizing compounds such as phalloidin and Jasplakinolide, which interfere with the correct maturing of F-actin and prevent its turnover (Pospich et al., 2020), do not inhibit root growth. Actin-depolymerizing drugs Latrunculin B and Cytochalasin, which sequester actin monomers or bind to the ends of F-actin, respectively, were shown to inhibit root growth (Baluška et al., 2001; Mancuso et al., 2006). Latrunculin B induces a reduction in F-actin density and an increase in bundling accompanied by a reduction in filament elongation rate and dynamics (Staiger et al., 2009; Rosero et al., 2013). Actin disruption by Latrunculin B treatment seems to influence cell elongation rather than cell division in roots of both *A. thaliana* and *Z. mays* (Baluška et al., 2001). Thus, in this case it is clear that the increase in bundling of actin is not directly related to promoting root growth, but that other mechanisms are also important. The myosin ATPase inhibitor 2,3-Butanedione 2-Monoxime (BDM) impacts the myosin-based actin cytoskeleton movement and also disrupted root growth in both *A. thaliana* and *Z. mays* (Šamaj et al., 2000; Mancuso et al., 2006). Interestingly, BDM impacted myosin-based actin movement mainly in the transition zone and beginning of the elongation zone, thus affecting the progression to cell elongation (see overview in Figure 1; Šamaj et al., 2000).

**Table 1:** Inhibitor effect on root growth, cell expansion, vacuolar shape, and actin organization and dynamics.

Inhibitor	General role	Effect on root growth	Effect on actin (cell type)	Effect on cell length (cell type)	Effect on vacuole shape (cell type)	References
Actin inhibitors						
Jasplakinolide	Actin stabilization (turnover inhibition)	None			Larger main vacuole (late meristem cells)	Mancuso et al., 2006 Scheuring et al., 2016
Phalloidin	Actin stabilization (turnover inhibition)	None				Mancuso et al., 2006
Latrunculin B	Actin depolymerization	Growth inhibition	Disruption of longitudinal actin arrays (EZ, maize)	Inhibition of cell elongation^*^	Round and smaller main vacuole (late meristem cells)	Baluška et al., 1997, 2001 Baluška et al., 2001 Mancuso et al., 2006 Scheuring et al., 2016
Cytochalasin D	Actin depolymerization	Growth inhibition		Cell length inhibition (AEZ, maize cortex)		Mancuso et al., 2006 Šamaj et al., 2000
2,3 Butadione (BDM)	Inhibition of myosin ATPase		Stabilization of AF – thicker bundles (maize^*^)	Cell length inhibition (TZ and AEZ, maize cortex)		Šamaj et al., 2000
Hormones						
Indole-3-Acetic-Acid (IAA)	Native auxin	Growth inhibition	Increased AF density and unbundling TZ/EZ	Cell elongation inhibition^*^		Rahman et al., 2007 Lanza et al., 2012 Arieti and Staiger, 2020
1-Naphtalene-Acetic-Acid (NAA)	Synthetic auxin	Growth inhibition		Cell elongation inhibitor^*^	Increased vacuolar constriction (late meristem cells)	Rahman et al., 2007 Scheuring et al., 2016
2,4-Dichlorophenoxyacetic acid (2,4-D)	Auxin analogue	Growth inhibition	Actin depolymerization^*^	Mild cell elongation inhibitor, cell proliferation inhibitor^*^		Rahman et al., 2007
24-epibrassinolide (eBL)	Brassinosteroid	Root waving	Finer, shorter, and more dynamic AF^*^			Lanza et al., 2012
trans-zeatin (t-zeatin)	Cytokinin	Growth inhibition	Decreased AF density, increased AF bundling; disrupted vertical AF arrangement TZ/EZ	Cell elongation (TZ)		Takatsuka et al., 2018 Kushwah et al., 2011

AF, actin filaments; TZ, transition zone; AEZ, apical elongation zone; and EZ, elongation zone.

* no data on cell type.

Cell growth has been proposed to be driven by a sequential coordination between cell wall modification and turgor pressure changes (Zimmermann et al., 1980). In this context, a turgor pressure increase is expected to raise the mechanical pressure on the cell wall, aiding cell elongation (Reviewed in Kaiser and Scheuring, 2020). The actin cytoskeleton has been demonstrated to be key component of cell wall remodeling, mediating vesicle transport of cellulose synthase (CESA) complexes, cell wall modifying enzymes (e.g., expansins) or non-cellulosic cell wall components (Baluška et al., 2002; Leucci et al., 2006; Crowell et al., 2009; Sampathkumar et al., 2013). It is thought that the actin cytoskeleton transports vesicles containing cell wall modifying components that promote cell elongation. This hypothesis is supported by the negative effect of Latrunculin B-mediated actin depolymerization on the dynamics of CESA-containing Golgi bodies in roots or the decreased delivery and uptake of pectins and other polysaccharide components of the cell wall observed in other plant tissues (Baluška et al., 2002; Leucci et al., 2006; Sampathkumar et al., 2013). Conversely, plant cells treated with the cell wall inhibitor isoxaben or mutants with decreased cellulose content show altered F-actin distribution (Tolmie et al., 2017; Huang et al., 2020).

Another important player in maintaining turgor pressure in plant cells is vacuoles. Vacuolar occupancy of the cell increases with and correlates with cell elongation (Dünser et al., 2019). In other plant cell models such as BY-2 cells or stomata guard cells, actin has been shown to play an important role in vacuolar remodeling (Higaki et al., 2006; Li et al., 2013). Actin inhibitor studies have demonstrated the importance of a fully functional actin cytoskeleton for vacuolar shaping during primary root cell elongation. In root epidermal late meristematic cells, actin stabilization (Jasplakinolide) results in larger main vacuoles while actin disruption (Latrunculin B) induces the appearance of a round and smaller main vacuole, especially in cells prior to rapid second cell elongation (Scheuring et al., 2016). However, Takatsuka et al. (2018) show that no striking vacuolar changes occur prior to the second elongation event, indicating that they do not seem to be its trigger.

In summary, longitudinally oriented actin bundles and increased actin dynamics are indicative of primary root cell elongation. Importantly, actin filaments seem to have a crucial role in the second rapid cell expansion of root cells. Why are these bundled and active filaments necessary for cell elongation in the root? Likely there are two important factors: First, actin filaments help to deliver cell wall remodeling components to the growing sides of the cell. There is a clear link between actin–myosin-mediated cytoplasmic streaming, endomembrane remodeling, and cell growth, suggesting that the organization of actin parallel to the growth axis could aid cell elongation by increasing delivery of endocytic cargoes (Peremyslov et al., 2012; Tominaga et al., 2013). Second, there is the plant-specific need to regulate a growing cells’ turgor pressure, which could be mediated by the vacuolar network. Root cell elongation is inversely correlated to vacuolar constriction and the two are tightly depending on specific actin organization and dynamics. However, the connection between vacuolar occupancy, actin, and root growth should be explored in more detail.

## The Role of Actin Isoforms and Actin-Binding Proteins in Primary Root Growth

The actin cytoskeleton consists of actin filaments made up of strands of G-actin polymers of different isoforms, depending on the cell type, tissue, or specific cellular function. Proteins that bind to, interact with, and modify actin filaments to coordinate their orientation and dynamics, are crucial for the operation of the actin cytoskeleton. Actin nucleators promote *de novo* or branched filament assembly; actin-capping/severing proteins regulate the dynamics of actin filaments by mediating its turnover; network protein (NET) family members link actin and the endomembrane system; myosin motor proteins facilitate actin-based vesicle and organ motility. In the next section, we dissect which particular isoforms of actin are crucial for root growth and we discuss how and why various actin-binding proteins affect root growth.

### Actin

G-actin is a highly conserved protein that is encoded by eight functional genes in plants. They can be classified, based on their sequence and temporal and spatial expression pattern, into two major groups: reproductive (ACT1, ACT3, ACT4, ACT11, and ACT12) and vegetative (ACT2, ACT7, and ACT8). The first class is expressed in pollen, ovules, and seeds while the second class is expressed in vegetative tissues, including some of the floral organs (e.g., petals, sepals, and carpel) and germinating seeds. All vegetative actins were observed to be strongly expressed in young roots (McDowell et al., 1996; Reviewed in Meagher et al., 1999, 2000; Šlajcherová et al., 2012). Of the vegetative actins, ACT7 seems most involved in shaping primary root growth, because *act7* mutants have severe root elongation phenotypes and wavier roots (Gilliland et al., 2003; Kandasamy et al., 2009). At the tissue level, *act7* had a reduction in cell number in the proximal meristem zone and an increase in cell number in the transition zone (Takatsuka et al., 2018). Actin organization analysis in *act7* showed a delayed onset of longitudinal actin bundling at the boundary between the elongation and transition zones as observed in wild-type plants. This points to ACT7 as the main player in the second rapid cell elongation (Table 2; Kandasamy et al., 2009; Takatsuka et al., 2018). The other two vegetative actin isoforms (ACT2 and ACT8) seem to have a more relevant role in root hair formation and only show root elongation phenotypes in dominant-negative mutants (*act2-2D* and *frz1*, respectively; Nishimura et al., 2003; Kandasamy et al., 2009; Kato et al., 2010). Only when both isoforms are knocked out, the phenotype of *act7* mutants could be mimicked, suggesting a secondary role of ACT2 and ACT8 in root cell elongation (Table 2) (Takatsuka et al., 2018). Consistent with phenotypical observations, *act7-4* and not *act2 act8* mutants show decreased vacuolar fragmentation in late meristematic epidermal cells, consistent with actin stabilization treatment by Jasplakinolide. This failure to regulate proper vacuolar constraining probably contributes to a delay in the initiation of fast cell elongation (Scheuring et al., 2016). The wavier root phenotypes observed in *act7-4* and *der1-2* (carrying an *ACT2* point mutation) mutants correlate with the higher presence of oblique cell walls, also pointing to a role of these two isoforms in cytokinesis (Gilliland et al., 2003; Vaškebová et al., 2018).

**Table 2:** Actin and actin-binding protein role in primary root growth.

Protein family	Contribution to root/root zone growth	Molecular function	References
Monomeric actin			
ACTIN ACTIN7 ACTIN2 and 8	Main effect on primary root growth and waving. Main actin involved in second rapid cell elongation (TZ-EZ boundary). Mild effect in primary root growth. Secondary role in cell elongation	Change of actin organization (e.g., bundling) Maintenance of vacuolar constriction in late meristem cells	Nishimura et al., 2003 Kandasamy et al., 2009 Kato et al., 2010 Scheuring et al., 2016 Takatsuka et al., 2018
Actin nucleators and actin crosslinking/bundling proteins			
ARP2/3 and SCAR/WAVE complex	Root growth promotion. Regulation of speed cell expansion at the EZ meristem cell division	Regulation of actin organization and cell wall material delivery	Dyachok et al., 2008, 2011 Takatsuka et al., 2018
FORMINS Class I FH1 FH4 FH5 FH8 Class II RMD	Regulation of root growth. Unknown, only expression data available Regulation of root growth Regulation of root growth Promotion of root growth. Regulation of root waving. EZ cell length control.	Developmentally regulated subcellular localization. Increase in actin dynamics and maintenance of low bundling. Maintenance of endomembrane dynamics Role in cell division (cytokinesis) Role in cell division (phragmoplast localization) Actin bundling and orientation	Deeks et al., 2005 Rosero et al., 2013 Oulehlová et al., 2019 Ingouff et al., 2005 Xue et al., 2011 Li et al., 2014
Actin capping/severing proteins			
VILLINS VLN2, VLN3, VLN4	Root directional growth	Facilitation of actin turnover and bundling	van der Honing et al., 2012 Zou et al., 2019
ADF/COFILINS D-type ADF1 ADF2	Root growth containment	Preventing actin bundling	Dong et al., 2001b Clément et al., 2009
AIP1	Root growth promotion	Decrease of actin bundling, increase of actin dynamics important for trafficking	Ketelaar et al., 2004 Dhonukshe et al., 2008
PROFILINS Vegetative PRF1 PRF3	Root growth promotion Root growth inhibition		Ramachandran et al., 2000 Fan et al., 2013
Actin-membrane binding proteins			
NET NET1A/B (Actin-PM) NET4A (Actin-Tonoplast)	Root growth promotion Root growth inhibition through vacuolar constriction in the MZ-TZ region		Deeks et al., 2012 Kaiser et al., 2019
Motor proteins			
MYOSINS Myosins XI-1, XI-2, and XI-K	Root growth promotion. EZ cell length control.	Increase of actin organization, density, and dynamics. Maintenance of vacuolar constriction in late meristem cells.	Šamaj et al., 2000 Peremyslov et al., 2010 Scheuring et al., 2016

TZ, transition zone; and EZ, elongation zone.

### Actin-Binding Proteins

#### Actin Nucleators and Actin Crosslinker/Bundling Proteins

Actin nucleators promote *de novo* or branched filament assembly from available actin monomers or profilin-bound G-actin. The actin nucleators most relevant to main root growth are the actin-binding proteins belonging to the ARP2/3 complex and formins. The ARP2/3 complex is formed by seven subunits that are ubiquitously expressed throughout plant development (Klahre and Chua, 1999; Le et al., 2003; Li et al., 2003; Mathur et al., 2003; El-Assal et al., 2004; Zhang et al., 2013a; García-González et al., 2020). Based on similarity to animal models, the ARP2/3 complex is hypothesized to participate in actin filament nucleation and branching. Although some of its subunits have been shown to colocalize with actin-branching sites, its function is yet to be demonstrated in planta (Fišerová et al., 2006). Other studies link the function of ARP2/3 to actin-microtubule interplay (Zhang et al., 2013a; Havelková et al., 2015; Cifrová et al., 2020). The lack of functional ARP2/3 complex subunits or its activators results in a reduction of primary root growth rate, accompanied by less cell divisions in the meristematic zone, more cells in the transition zone, and a shorter elongation zone (Dyachok et al., 2008, 2011; Takatsuka et al., 2018). The ARP2/3 complex requires upstream activation by the SCAR/WAVE complex (Reviewed in Yanagisawa et al., 2013). The SCAR/WAVE complex subunits BRK1 and SCAR1 were observed in the root tip epidermis up until the elongation zone, mainly in three-way cell junctions. *brk1* and *arp3* mutants show disorganized actin arrays in the elongation zone (Dyachok et al., 2011). Furthermore, the SCAR/WAVE complex seems to be involved in cell wall deposition in three-way junctions of root epidermal cells (Dyachok et al., 2008). Taken together, the actin pool coordinated by the ARP2/3 complex and its activators participate in the second rapid cell expansion at the boundary of the transition and elongation zones, presumably through its contribution to correct cell wall modification (Table 2).

Formins are actin-binding proteins responsible for *de novo* actin nucleation, actin filament elongation and bundling. In angiosperms, two groups of formins exist, encoded by a total of 21 genes. Typically, class I formins are associated with membranes, while class II are cytoplasmic (Grunt et al., 2008; Reviewed in Cvrčková, 2012, 2013). The knowledge on the role of formins in root growth is scarce; however, it indicates a role in vesicle trafficking and endomembrane remodeling (Table 2). Class I formins *FH1*, *FH4*, and *FH8* are expressed in roots (Deeks et al., 2005; Oulehlová et al., 2019). In particular, FH1 subcellular localization has been reported to vary according to the developmental stage in epidermal root cells. In the meristematic zone, FH1-GFP accumulates to cell plates of dividing cells and anticlinal walls. In the transition zone, plasmodesmata and tonoplast-associated FH1-GFP can be observed, while the signal is progressively lost toward the elongation and maturation zone. A free, mobile fraction of FH1-GFP dots could be observed throughout root cell development, which are likely associated with late endosomes (Oulehlová et al., 2019). Although *fh1* plants do not show a clear root elongation phenotype unless treated with Latrunculin B, a phenomenon very likely due to functional redundancy of formins. Latrunculin B treatment causes an increase in bundling and a decrease in actin dynamics, especially in the differentiation zone (Rosero et al., 2013). Microtubule dynamics are also affected in the *fh1* mutant, indicating again the importance of the crosstalk between actin and microtubules (Rosero et al., 2013). Furthermore, these data suggest a role of FH1 in regulating root cell elongation through the maintenance of endomembrane system dynamics. *FH8* is expressed mainly in the root meristem, and its subcellular localization is in the nucleus in interphase cells and in the phragmoplast during cytokinesis, suggesting a role of FH8 in cell division. *fh8* mutant plants have no reduced root growth phenotype, except for an increased sensitivity to Latrunculin B treatment (Xue et al., 2011), similar to the *fh1* mutant. The absence of a clear phenotype in *fh8* mutants does not exclude the possibility of a role of this formin in root growth, as suggested by expression data. Another class I formin, FH5, has also been observed to participate in cytokinesis in root meristematic cells (Ingouff et al., 2005). These cases highlight the importance of breaking genetic redundancy in these sizeable protein families. Class II formins have been less studied regarding root growth. A double null mutant allele of the RMD formin has shorter elongation zone cells accompanied by an increased amount of transversally arranged actin bundles. This results in a wavy root phenotype and reduced root growth rate (Li et al., 2014). In the double RMD formin mutant, increased persistence of FM4-64-labeled and OsPIN2 BFA bodies after treatment washout showed that endomembrane vesicle trafficking was impaired in this mutant (Li et al., 2014).

#### Actin-Capping/Severing Proteins

Villins are versatile calcium-responsive actin-binding proteins that can modify actin dynamics by depolymerizing, capping, severing, and bundling actin filaments. *Arabidopsis thaliana* encodes five villin paralogs which are widely expressed (Klahre et al., 2000; Khurana et al., 2010; Zhang et al., 2010; Reviewed in Huang et al., 2015). Although no root elongation phenotypes have been reported for villin mutants, VLN2, VLN3, and VLN4 have been shown to be facilitators of actin turnover and bundling in epidermal root cells (Table 2). The double mutant *vln2 vln3* shows twisted roots and *vln4* has altered root bending upon gravistimulation (van der Honing et al., 2012; Zou et al., 2019). It is possible that higher-order mutants are needed to unveil a role of villins in primary root length determination, but the above-mentioned defects in root bending and twisting of villin mutants indicates that they are required for the fine tuning of root directional growth (van der Honing et al., 2012; Zou et al., 2019).

ADF/cofilins are a family of actin-binding proteins that promote depolymerization, severing and bundling of actin filaments. There are 11 ADF genes that can be divided into two subfamilies: D-type ADFs with depolymerizing activities (ADF1-4, ADF6-8, and ADF10-11) and B-type ADFs with bundling activity (ADF5 and ADF9; Nan et al., 2017). Expression analysis of the members of this family has shown that all ADFs except *ADF7* and *ADF10* are expressed in root tissues of which *ADF1*, *ADF5*, and *ADF6* in the vasculature, *ADF5* and *ADF9* in the RAM and *ADF8* and *ADF11* in trichoblasts (Dong et al., 2001a; Kandasamy et al., 2007; Ruzicka et al., 2007). *ADF1* overexpression induced shorter roots, consistent with the phenotype observed when plants are treated with actin-depolymerizing drugs such as Latrunculin B. In contrast, reduced expression of *ADF1* resulted in longer roots. Research in hypocotyls showed an increase of actin bundling in mutants with decreased *ADF1* expression (Dong et al., 2001b). *ADF2-RNAi* lines show increased actin density and bundling in roots but no visible root phenotype other than an occasional reduction in root apical meristem size, similar to the effect obtained with the actin-stabilizing drug Jasplakinolide (Clément et al., 2009). Once more, *ADF* genetic redundancy may be the key to the lack of observable root phenotypes in knockout mutants, but the increase in actin bundling and density in the respective mutants suggests that ADF1 and ADF2 specifically are involved in increasing actin turnover in root epidermis. This is a hint of their function in root elongation; however, more research is needed to uncover the importance of other ADFs in this process.

ACTIN INTERACTING PROTEIN 1 (AIP1) is facilitating the activity of ADF/cofilin proteins and has a function in capping the barbed end of actin filaments (Ketelaar et al., 2004). Consistent with the effect observed by drugs like Cytochalasin D, *AIP1-RNAi* lines show reduced root growth accompanied by a general increase in actin bundling (Ketelaar et al., 2004). Downregulation of *AIP1* expression leads to reduced FM4-64 internalization comparable to the effect observed after Jasplakinolide treatment. The phenotype observed is possibly a consequence of actin filament stabilization (Dhonukshe et al., 2008).

Profilins regulate the pool of available G-actin to inhibit *de novo* polymerization and the addition of new monomers to existing filaments; they also contribute to depolymerization and monomer recycling (Michelot et al., 2005; Reviewed in Blanchoin et al., 2010). Profilins belong to a multigene family and can be divided into two main groups: vegetative (PRF1–PRF3) and reproductive (PRF4 and PRF5; Kandasamy et al., 2002). The vegetative profilin gene *PRF1* has higher promoter activity in the elongation zone (Ramachandran et al., 2000). Analysis of *prf1* knockout lines resulted in contradicting root length phenotypes that overall indicate a role for PRF1 in regulating root growth (Ramachandran et al., 2000; McKinney et al., 2001; Müssar et al., 2015; Cao et al., 2016). The *prf3* mutant has no observable root growth phenotype and overexpression of PRF3 induces either WT-like or shorter roots (Fan et al., 2013; Müssar et al., 2015). PRF1 was shown to maintain actin filament density in elongation zone root epidermal cells (Cao et al., 2016). Analysis of actin dynamics in hypocotyl cells suggests a role of PRF1 in the positive regulation of actin turnover through the facilitation of nucleation of other actin pools through formin activity (Cao et al., 2016). Though, it is likely that different profilins regulate the dynamics of distinct pools of actin, therefore producing different phenotypes.

#### Actin-Membrane Binding Proteins

The network (NET) family of proteins are linkers between actin and the endomembrane system. They are characterized by the presence of a conserved F-actin-binding domain in the N-terminal region. Several members of this family have been described to connect the actin cytoskeleton to different membrane compartments; however, the exact mechanism of NET proteins action to modulate the actin cytoskeleton remains to be determined (Deeks et al., 2012; Reviewed in Wang and Hussey, 2015). NET1A and NET1B connect F-actin to the plasma membrane and plasmodesmata. Both are expressed in the root meristem and early elongation zone and the double knockout mutant *net1a net1b* has shorter roots (Deeks et al., 2012). NET4 is localized to the tonoplast and is also expressed in the root meristem and early elongation zone. While *net4a net4b* show no root growth phenotype, *NET4A* overexpression induces shorter roots. Both mutants display higher cellular vacuolar occupancy. However, *net4a net4b* show a more fragmented vacuolar network than NET4A-GFP^OE^, reminiscent of the effect obtained by Latrunculin B and Jasplakinolide treatment (e.g., depolymerization and stabilization of actin, respectively). Increase in NET4 expression leads to more constricted vacuoles and failure to establish the onset of cell elongation. Higher amounts of NET4 are present in the late meristematic/transition zone, correlating with regions of high vacuolar constrictions (Scheuring et al., 2016; Kaiser et al., 2019). These results again indicate the impact of actin on vacuolar occupancy and consequently on root growth.

#### Motor Proteins

Myosins are actin-binding motor proteins with multiple roles, mainly known for their role in vesicle and organelle motility. Increasing evidence also points to their involvement in the control of actin organization and dynamics. Two myosin families have been described in angiosperms: myosin VIII, which are found at the cell cortex presumably creating tension through actin, and myosin XI which are intracellular and colocalize with several organelles, some of unknown identity (Reviewed in Ryan and Nebenführ, 2018). Triple myosin mutants in *Arabidopsis* (*xi-k/1/2*, *xi3ko* for short) have reduced hypocotyl and root growth. Specifically, roots have shorter cells at the elongation zone accompanied by a less dense, more bundled, and randomly oriented actin. This coincides with a failure of the *xi3ko* mutant to coordinate constriction of the vacuole in late meristem cells, similar to the above-mentioned *act7-4* mutant or the NET4-GFP^OE^ overexpressing mutants (Scheuring et al., 2016; Kaiser et al., 2019). Also, actin dynamics are slowed down with less severing frequency which probably leads to a reduced vesicle delivery (Peremyslov et al., 2010; Reviewed in Zhu and Geisler, 2015). This correlates with the results obtained in previous studies using the myosin inhibitor BDM and further confirms the importance of myosin XI in regulating actin dynamics and cell elongation (Šamaj et al., 2000).

In summary, all the components of the actin network, including actin monomers themselves, have a high degree of functional redundancy and functional specialization. Therefore, it is not always straightforward to draw conclusions on their effects on the regulation of root growth. However, in many cases overexpression, silencing, or knockout of actin components seems to reduce root growth, except for ADF1 knockout, which increases root length. This suggests that disrupting the actin network often has a detrimental effect, considering that different actin-modifying proteins have various effects on actin organization (see Table 2). However, superficially similar effects can also be masked by effects of actin organization in vacuolar size and occupancy, or by effects on hormone trafficking, which will be discussed in the next section.

## Hormonal Regulation of Actin-Mediated Primary Root Growth

Primary root growth plasticity depends on the very tight balance between cell division, cell elongation, and cell differentiation. The rapid adjustment to new stimuli is mediated by the coordinated action of a complex network of plant hormones. However, as the previous sections showed, the actin cytoskeleton also plays an important role here. Often hormones lead to changes in actin, and actin changes lead to differences in hormone distribution. In this section, we focus on the role of the actin cytoskeleton in hormonal control of primary root growth, and especially the role of auxin, the most-studied hormone in this context.

### The Auxin-Actin Feedback Loop Modulates Root Development

Auxin is the most studied and important plant hormone in relation to the regulation of the actin network in the context of main root growth. Changes in the actin network have a strong effect on polar auxin transport. Conversely, auxin has a significant effect on the actin cytoskeleton itself (Reviewed in Zhu and Geisler, 2015; Zhu et al., 2016; Sahi et al., 2018; Zou et al., 2019; Arieti and Staiger, 2020). The actin cytoskeleton response to auxin has been predominantly analyzed in the root epidermis transition and elongation zones, as this hormone is strongly involved in root cell elongation during root growth and tropic responses. Actin inhibitor studies have shown that Cytochalasin D or Latrunculin B treatment alters the plasma membrane distribution of the auxin efflux carriers PIN1 and PIN3, which are key regulators of tropic growth (Geldner et al., 2001; Friml et al., 2002). Conversely, auxin treatment is known to inhibit root growth and cell elongation (Table 1; Evans et al., 1994; Rahman et al., 2007; Fendrych et al., 2018). Initial studies with the native auxin Indole-3-Acetic-Acid (IAA) reported contradicting effects on actin filament bundling (Rahman et al., 2007; Nick et al., 2009; Lanza et al., 2012; Takahashi et al., 2017). The differences between these studies suggest that precise concentration, duration, form of auxin, and root cell developmental status appear to be an important driver of actin changes. However, detailed quantitative analysis of IAA treatment reported a significative increase in density, unbundling, and higher organization of actin filaments in elongating cells of the root, which is generally maintained even after 60 min of treatment (Figure 1; Table 1; Lanza et al., 2012; Arieti and Staiger, 2020). These changes in actin organization, induced by IAA treatment, are consistent with the general trend that less bundled actin filaments are associated with less cell elongation. The synthetic auxin 1-Naphtalene-Acetic-Acid (NAA) was suggested to inhibit root growth through the actin-mediated increase of vacuolar constriction in late meristematic cells. Consistent with this hypothesis, vacuoles of plants pre-treated with actin-disrupting drugs show less sensitivity to external auxin (Table 1; Scheuring et al., 2016). The auxin analog 2,4-Dichlorophenoxyacetic acid (2,4-D) affects root growth through the inhibition of both cell division and cell elongation (Table 1; Rahman et al., 2007). Unlike IAA, 2,4-D effects mimic those of the actin-depolymerizing drug Latrunculin B (Figure 1). 2,4-D treatment disturbs auxin signaling via a different ubiquitin ligase complex than IAA, the SMAP1-SCF^TIR1^ complex, thereby affecting post-translational modifications of actin (Rodríguez-Serrano et al., 2014; Takahashi et al., 2017). Each auxin analog employed in the above-mentioned studies differentially affects auxin signaling, which allows us to pinpoint the molecular mechanism of auxin-dependent actin responses (Ma and Robert, 2014).

Auxin can also affect the actin cytoskeleton via the activation of transcription: promoter activity of the vegetative actin *ACT7* and transcription of several subunits of the actin nucleator ARP2/3 complex are enhanced by auxin treatment (Kandasamy et al., 2001; García-González et al., 2020). A feedback loop exists between auxin signaling and the actin cytoskeleton: treatment with the auxin transport inhibitor naphthylphthalamic acid (NPA) inhibits root growth by reducing cell division rate and induces a reduction in actin filament density (Rahman et al., 2007). In hypocotyls, NPA treatment leads to an increase in actin filament bundling and a decrease in actin bundle density in hypocotyl cells, without disturbing long actin filaments. Plants lacking the actin isoform ACT7 (*act7-4*) are insensitive to NPA treatment (Zhu et al., 2016). Moreover, actin seems to be required for the correct expression, localization, and trafficking of auxin carriers. *act7-4* mutants have reduced expression of ABCB transporters, altered localization of PIN1 and PIN2, and increased endosomal retention of auxin transporters (Zhu et al., 2016). The wavy phenotype of *act2-5* lines is connected to the delocalization from the plasma membrane of PIN2 (Lanza et al., 2012). When treated with Latrunculin B, PIN2 accumulates into intracellular vesicles and disappears largely from the plasma membrane (Kleine-Vehn et al., 2009; Glanc et al., 2019). These results indicate the importance of the actin network on the trafficking of hormone transporters. Other actin-binding protein mutants, such as the rice class II formin RMD and VILLIN4, show reduced polar auxin transport in roots (Li et al., 2014; Zou et al., 2019). Furthermore, the expression of the above-mentioned *RMD* gene in rice is facilitated by the action of the TIR/AFB1 pathway-dependent OsARF23 and OsARF24 (Li et al., 2014). Further work revealed that the auxin influx carrier AUX1 was necessary to mediate the actin filament response triggered by auxin treatment in roots (Arieti and Staiger, 2020).

It appears that auxin and actin are very much interlinked via the control of polar auxin transport by actin dynamics, or via the direct or transcriptional effects of auxin on the same actin dynamics. Exactly how these two important players feedback on each other and create coordinated development and tropisms is still a topic with many questions. Especially interesting is the mechanistic connection between the intracellular transport of vesicles containing auxin transporters and the status of the actin network, or the way in which auxin sensing is quickly translated into changes in the actin network.

### Other Hormones

Besides auxin, other hormones have been connected to actin organization during primary root growth, although they have been studied with less depth. For instance, brassinosteroid treatment induces a wavy phenotype that correlates with an increase of finer, shorter and more dynamic actin filaments in epidermal cells (Lanza et al., 2012). The observed phenotypes are comparable to those resulting from *ACT2* mutation. Furthermore, both brassinosteroid treatment and *act2-5* mutation result in PIN2 delocalization in the root epidermis. This suggests that brassinosteroids, auxin, and actin have a shared pathway regulating root waving (Table 1; Lanza et al., 2012).

Cytokinin treatment decreases root growth via a reduction in meristem size (Ioio et al., 2007; Růzǐčka et al., 2009; Takatsuka et al., 2018). Cytokinin has a negative effect on auxin signaling through its inhibition in the transition zone (Figure 1; Reviewed in Vanstraelen and Benková, 2012; Takatsuka and Umeda, 2014; Wybouw and De Rybel, 2019). The response to cytokinin is mediated through the modulation of the actin cytoskeleton at the boundary between the transition and elongation zone, as observed by an increase in actin bundling and a decrease in actin filament density after treatment (Takatsuka et al., 2018). Also, an increase in cytokinin was shown to disrupt the longitudinal rearrangement of actin filaments in roots, in agreement with the tendency of longitudinally organized actin bundles to induce root growth (Kushwah et al., 2011).

The actin cytoskeleton seems to be a nexus in mediating the complex responses to auxin during root growth. Generally, auxin tends to induce more longitudinally organized and less bundled filaments, while cytokinin tends to induce an increase in bundling and less organized actin filaments. Current models suggest that there is a cytokinin-dependent generation of an auxin minimum starting at the transition zone (Reviewed in Rutten and Tusscher, 2019). This hormone gradient correlates with the start of cell elongation and the increase in actin organization toward a more bundled and longitudinally oriented network in the elongation zone. Failure to generate the proper hormone gradients results in growth inhibition. Exactly how hormone gradients lead to changes in actin filament organization is not very clear. In the case of auxin and cytokinin it is likely auxin, which has the terminal effect, while cytokinin controls the auxin gradient. This notion could be tested by tissue-specific inhibition of cytokinin signaling to disturb the auxin gradient. However, only the systematic and comparative analysis of actin organization in specific cell types in response to the manipulation of hormonal pathways will unveil the existing interdependence in cytoskeletal control. Furthermore, the involvement of specific actin-binding proteins downstream of hormone signaling would contribute to understanding which actin organization and dynamics modifications are more relevant. Auxin and cytokinin are also very important for the regulation of root tropisms. This is the stage for another interaction between hormones and actin, which we will discuss next.

## Actin Control of Primary Root Tropisms and Light Response

Root plasticity relies on the ability of the root to direct its growth toward or against a stimulus. For the primary root tip this is achieved mostly through asymmetric cell expansion that allows for its bending. Mainly two tropisms have been studied in relation to actin: positive gravitropism and negative phototropism. In both cases actin is involved in both stimuli perception and response.

### Gravitropism

Primary root directional growth is a multistep process that goes from gravity sensing to signal transduction and response execution. The gravity vector is sensed by sedimenting statoliths which results in the relocalization of PIN auxin transporters and a differential auxin gradient, causing differential growth of cells between top and bottom of the root tip (Reviewed in Su et al., 2017). Statoliths are amyloplasts functioning in gravity perception and sediment in the central columella cells when the root orientation changes. This sedimentation has been recognized as the primary process controlling gravity perception (Sack, 1997; Reviewed in Nakamura et al., 2019). A fine network of F-actin surrounds statoliths in central columella cells. The results from actin cytoskeleton inhibitors and knockout mutants of the actin network components show that F-actin is involved in the regulation of statolith repositioning (Figure 1; White and Sack, 1990; Baluška and Hasenstein, 1997; Volkmann et al., 1999; Voigt et al., 2005; Reviewed in Blancaflor, 2013). Actin disruption by Latrunculin B or Cytochalasin D and the inhibition of myosin activity by BDM enhance root tip bending to gravity, while the actin-stabilizers Jasplakinolide or phalloidin inhibit the gravitropic response (Hou et al., 2003; Li et al., 2005; Mancuso et al., 2006). Latrunculin B treatment results in faster statolith relocalization which indicates that the fine actin network fine-tunes statolith movement and repositioning after gravistimulus (Hou et al., 2003; Zheng et al., 2015). To date, the only actin-binding protein that has been connected to statolith sedimentation and PIN relocalization during the root gravitropic response is the ARP2/3 complex (Reboulet et al., 2010; Zheng et al., 2015; Zou et al., 2016). Knock-out of the ARP2/3 subunit ARP3 leads to a slower gravitropic response which is associated with reduced statolith sedimentation and an increase in actin bundling around statoliths (Reboulet et al., 2010; Zheng et al., 2015; Zou et al., 2016). The E3 ubiquitin ligase SGR9 mediates at least part of the interaction between the actin network and statoliths. Plants lacking SGR9 show impaired statolith sedimentation resulting in a reduced gravitropic response, accompanied by an abnormal actin filament network around statoliths. This phenotype could be rescued by the application of the actin-depolymerizing drug Latrunculin B, suggesting a role of SGR9 in releasing statoliths from the actin cytoskeleton through its interactors (Nakamura et al., 2011). These experiments studied the statoliths in the endodermis of the inflorescence stem and not in roots. However, the mechanism of gravity perception between roots and shoots is likely to be very comparable at the statolith level. Further work should elucidate whether the same mechanism exists in roots. Additionally, it would be interesting to know which actin-binding proteins interact with SGR9. Experiments with decapped roots, laser ablation of columella cells, starch-less mutants, or maintaining the root cap at a specific angle suggest an alternative gravisensing mechanism at the distal elongation zone, out of the root cap (Wolverton et al., 2002, 2011; Mancuso et al., 2006). Our combined knowledge points to a significant role of actin in gravity perception via the mediation of statolith sedimentation. However, the actin cytoskeleton has also been proposed to mediate PIN3 relocalization to the lateral plasma membrane in columella cells upon gravistimulation. PIN3 clathrin-mediated endocytosis is naturally increased when seedlings are re-oriented, a phenomenon that can be enhanced by Latrunculin B treatment (Friml et al., 2002; Kleine-Vehn et al., 2010). This represents a connection between actin and gravity signal transduction through the regulation of the asymmetric redistribution of auxin. It is likely that actin is involved in the delivery of endocytic cargoes containing PIN3 to the plasma membrane. Further downstream, actin bundles could mediate the asymmetric growth of the root meristem during the gravitropic response. Although a change toward more oblique oriented microfilaments has been observed in stele cells upon gravistimulation, no clear correlation between actin organization in epidermal cells and root asymmetric growth has been determined (Blancaflor, 2002; Pozhvanov et al., 2013). Evidence in support of a direct role of actin in the asymmetric cell growth of the root tip is the observations that vesicle trafficking is defective in root epidermal cells of various actin and actin-binding protein mutants (Li et al., 2014; Mao et al., 2016; Zou et al., 2016; Oulehlová et al., 2019). The process of root-negative phototropism, where the root tip bends away from a blue or red light source, is also partly regulated via actin, since it is regulated via the same auxin transporters as gravitropism (Wan et al., 2012; Zhang et al., 2013b). In summary, actin has a role in the sedimentation and repositioning of statoliths which determines the strength of gravity perception. It is not yet clear to what extent the F-actin network is responsible for relaying the mechanical signal of sedimenting statoliths to a differential PIN distribution and gravitropic growth.

### Effects of Light on Actin Organization in Roots

The processes of gravitropism and root growth in general are heavily influenced by light perception (Silva-Navas et al., 2016). When a seedling is exposed to light, hypocotyl growth is reduced, cotyledons open and make chlorophyll, while root length growth strongly increases. Seedlings growing fully in darkness have a strongly reduced root growth (Sassi et al., 2012). Actin filaments in elongating root cells are normally vertically oriented and bundled. However, in darkness, actin oriented seemingly randomly, with less bundling, and less alignment (Dyachok et al., 2011). Furthermore, it has been shown that PRF1 is important for root growth and development during photomorphogenesis (McKinney et al., 2001). Even though roots normally grow covered from light in the soil, *Arabidopsis* roots express all types of common photoreceptors in plants, including the Far-Red light sensor PHYA (Reviewed in van Gelderen et al., 2018). Seedling roots grown in Far-Red light have a bundled actin organization, mostly resembling growth in white light, but in a *phyA* mutant, actin organization was more resembling that of dark-grown seedlings (Dyachok et al., 2011). This is an example of a direct link between light perception and actin organization in roots. However, most studies focus on the effects of light quality and quantity in above ground tissues, such as the hypocotyl epidermis (Henty et al., 2011) or stomatal guard cells (Iwabuchi et al., 2010). Current standard growth conditions keep roots exposed to light, but roots have not evolved to grow exposed to light and therefore efforts have been made in the past years to grow *Arabidopsis* roots in the dark, while keeping the shoot in light. A notable example of this is the D-root system, which is simply a dark cover around a square agar plate to shield the root from light, with an added insert at the shoot-root boundary (Silva-Navas et al., 2015). This system showed that covering the roots leads to increased root growth and less free radical production compared to the roots exposed to light (Silva-Navas et al., 2016). Using an alternative root shading method to D-root it was demonstrated that components of the SCAR/WAVE-ARP2/3 system BRK1 and SCAR1 are involved in regulating root growth in covered roots (Dyachok et al., 2011). The subcellular localization of BRK1-YFP changes from occurring at three-way junctions of cells to cytoplasmic upon darkness. SCAR1 is reduced in protein extracts from both soluble and membrane extract, and the central light regulator COP1 is involved in SCAR1 degradation (Dyachok et al., 2011). Therefore, it seems clear that actin has a significant role to play in the regulation of root growth by light. However, very little work has been done until now to further explore this connection. It is also of especial importance to do this in systems such as D-root to simulate the light conditions of the soil in order to obtain results that are relevant for plant growth in field or natural conditions.

## Concluding Remarks and Future Perspective

The actin network has been studied for a long time and there is a relatively large body of earlier work on the role of actin in regulating root growth. In the recent 5 years, this work has been revisited and expanded upon (Abu-Abied et al., 2018; Takatsuka et al., 2018; Oulehlová et al., 2019; Arieti and Staiger, 2020; de Bang et al., 2020). Although this sometimes led to contradictory or incomplete findings, the actin network is essential for root growth by regulating cell elongation and division. A main conclusion is that actin organization is developmentally regulated in the growing root tip. Bundled and longitudinally oriented actin filaments are a hallmark of cell elongation. Failure to achieve this arrangement by tampering with hormonal homeostasis or altering actin-binding protein function results in root growth defects. Most results with hormone and drug treatments and actin-binding protein mutants seem to correlate well with this general principle. These changes in actin organization affect cell growth in different ways, and of particular interest for further study is how actin-mediated delivery of cell wall modifying components aids cell elongation of the primary root meristem. There are also causal links between actin, vacuolar morphology, and cell elongation. However, more evidence is needed to show that defects in actin directly cause defects in vacuolar morphology that subsequently cause root growth defects.

Knowledge in this field is lacking evidence on the role of actin-binding proteins in regulating the actin network with regard to growth and development of the root meristem. In general, actin-binding proteins are well studied, however, with respect to root growth, there is little data available on phenotypic effects, usually due to a high degree of genetic redundancy. Overexpression often leads to detrimental effects on root growth, while single or even double mutants do not. With recent advances in CRISPR multiplexing, this redundancy problem can be tackled efficiently and the roles of actin-binding proteins in root growth and development can and should be elucidated.

Hormonal regulation of primary root growth occurs mainly through the coordination between auxin and cytokinin signaling, which contributes to the generation of developmental regions with a specific actin organization. The role of auxins, cytokinins, and especially other hormones in the control of the actin cytoskeleton requires more future work. This work would be made easier by the consistent and more precise denomination of which root zones and cell layers are analyzed, in order to compare the effects of individual hormones and their combination in the control of the actin network. Related to this is the study of root gravitropism, where actin has several different functions. It is involved in the direct sensing of gravity, controls the propagation of the hormone signal and it is likely involved in the subsequent regulation of cell elongation. This study brings together three different roles for actin and is therefore a very interesting subfield, which will benefit by recent advances in live imaging. With vertically oriented confocal laser scanning microscopes it is possible to study root gravitropism *in vivo* in real time and see details and subtleties not imaged before. Another new way of studying actin will be to use new optogenetic tools that allow the direct manipulation of the cytoskeleton or cytoskeletal components (Zhang et al., 2021), which might provide a deeper understanding of how actin functions in regulating cell elongation in complex tissues such as the root. With these new possibilities it will be possible to subtly disturb the actin network while imaging it at the highest temporal and spatial resolution.

## Author Contributions

All authors listed have made a substantial, direct and intellectual contribution to the work, and approved it for publication.

## Funding

This work was funded by the Czech Science Foundation (19-13375Y; JG-G) and the Netherlands Organisation for Scientific Research Vici grant 865.17.002 to Ronald Pierik (KG).

## Conflict of Interest

The authors declare that the research was conducted in the absence of any commercial or financial relationships that could be construed as a potential conflict of interest.

## Publisher’s Note

All claims expressed in this article are solely those of the authors and do not necessarily represent those of their affiliated organizations, or those of the publisher, the editors and the reviewers. Any product that may be evaluated in this article, or claim that may be made by its manufacturer, is not guaranteed or endorsed by the publisher.
